# A prospective investigation of developmental trajectories of psychosocial adjustment in adolescents facing a chronic condition - study protocol of an observational, multi-center study

**DOI:** 10.1186/s12887-021-02869-9

**Published:** 2021-09-14

**Authors:** Petra Warschburger, Ann-Christin Petersen, Roman E. von Rezori, Friederike Buchallik, Harald Baumeister, Reinhard W. Holl, Kirsten Minden, Annabel S. Müller-Stierlin, Christina Reinauer, Doris Staab, COACH consortium

**Affiliations:** 1 Department of Psychology, Counseling Psychology, University of Potsdam, Karl-Liebknecht-Str. 24-25, 14476 Potsdam, Germany; 2 Department of Clinical Psychology and Psychotherapy, Faculty of Engineering, Computer Science and Psychology, Institute of Psychology and Education, Ulm University, Ulm, Germany; 3 Institute of Epidemiology and Medical Biometry, ZIBMT, Ulm University, Ulm, Germany; 4 Charité University Medicine Berlin, Berlin, Germany; 5 German Rheumatism Research Centre, Berlin, Germany; 6 Department of Psychiatry and Psychotherapy II, BKH Günzburg, Ulm University, Günzburg, Germany; 7 Department of General Pediatrics, Neonatology and Pediatric Cardiology, University Hospital Düsseldorf, Düsseldorf, Germany; 8 Department of Pediatric Pulmonology, Immunology and Intensive Care Medicine, Charité University Medicine Berlin, Berlin, Germany

**Keywords:** Chronic conditions, Adolescents, Prospective, Quality of life, Resiliency, Coping, Protective factors, Type 1 diabetes, Juvenile idiopathic arthritis, Cystic fibrosis

## Abstract

**Background:**

Relatively little is known about protective factors and the emergence and maintenance of positive outcomes in the field of adolescents with chronic conditions. Therefore, the primary aim of the study is to acquire a deeper understanding of the dynamic process of resilience factors, coping strategies and psychosocial adjustment of adolescents living with chronic conditions.

**Methods/design:**

We plan to consecutively recruit *N* = 450 adolescents (12–21 years) from three German patient registries for chronic conditions (type 1 diabetes, cystic fibrosis, or juvenile idiopathic arthritis). Based on screening for anxiety and depression, adolescents are assigned to two parallel groups – “inconspicuous” (PHQ-9 and GAD-7 < 7) vs. “conspicuous” (PHQ-9 or GAD-7 ≥ 7) – participating in a prospective online survey at baseline and 12-month follow-up. At two time points (T1, T2), we assess (1) intra- and interpersonal resiliency factors, (2) coping strategies, and (3) health-related quality of life, well-being, satisfaction with life, anxiety and depression. Using a cross-lagged panel design, we will examine the bidirectional longitudinal relations between resiliency factors and coping strategies, psychological adaptation, and psychosocial adjustment. To monitor Covid-19 pandemic effects, participants are also invited to take part in an intermediate online survey.

**Discussion:**

The study will provide a deeper understanding of adaptive, potentially modifiable processes and will therefore help to develop novel, tailored interventions supporting a positive adaptation in youths with a chronic condition. These strategies should not only support those at risk but also promote the maintenance of a successful adaptation.

**Trial registration:**

German Clinical Trials Register (DRKS), no. DRKS00025125. Registered on May 17, 2021.

**Supplementary Information:**

The online version contains supplementary material available at 10.1186/s12887-021-02869-9.

## Background

Having a chronic condition (CC) means facing an additional stressor in life. Especially during adolescence, youths are confronted with a range of developmental tasks, e.g., finding a partner, preparing for future family and work life, accepting their own body, which can strongly interfere with the demands of a CC [1]. There is consistent evidence that facing a CC, irrespective of the specific diagnosis, increases the risk for mental health problems, such as anxiety and depression [2, 3]. But focusing only on mental disorders does, on the one hand, not cover the entire spectrum of potential functional limitations experienced in daily life. On the other hand, it neglects potential successful psychosocial adjustment. Including health-related quality of life (HRQoL) as a multifaceted, subjective indicator overcomes these shortcomings and allows getting a more concise picture of the psychosocial situation of these youths. Numerous studies have shown that HRQoL in chronically ill children and adolescents is impaired with small to moderate effect sizes compared to healthy youths [4–6]. Yet, CC as a risk factor is not inevitably associated with a poor adjustment. Considerable interindividual differences can be observed and it seems that the predominant response to the CC is a good adjustment [7]. This leads not only to the question of which characteristics and which processes are involved in maladjustment but also to the question of how to explain and attain a good adjustment. Up to now, the majority of research aims to explain the adverse outcomes and little is known about the emergence and maintenance of positive outcomes in the field of CC. There is evidence that higher age, being female, and higher CC severity are significantly associated with worse HRQoL [4–6] and increased mental health problems [8–10]. These factors only explain a small proportion of the variance, whereas psychological factors, e.g., coping or self-esteem, play a more significant role [1, 3, 7, 11–13]. Within the resilience-in-illness and the positive development framework, a number of resiliency factors, primarily addressed in the context of protective factors in CCs, promote positive adjustment via enhancing positive coping strategies and minimizing the influence of risk factors [14, 15]. Hereby, resiliency refers to the “… ability to cope with life circumstances in a positive way, even under conditions overshadowed by risk, and to develop appropriate coping skills despite unfavorable circumstances” ([16], p. 127). In the context of a CC, high levels of self-esteem, optimism, or self-efficacy proved to be relevant resiliency attributes [14, 17–19]. In addition, the so-called “secondary coping strategies” (focusing on efforts to adapt to the CC), e.g., acceptance, cognitive restructuring, or positive thinking, are considered beneficial [11, 20, 21]. It is well-known that resiliency and coping vary with developmental stage [11, 22] and there is first evidence for a dynamic and reciprocal relationship with HRQoL and mental disorders [23]*.* To sum up, prospective cohort designs are needed to understand the dynamic nature of adjustment in youths with a CC since outcomes as well as its protective and risk factors are not stable but fluctuate over time [11]. In addition, it is necessary to include sensitive developmental transition periods (entry into adolescence; emerging adulthood) with their manifold changes [11].

## Objectives

**Fig. 1:**
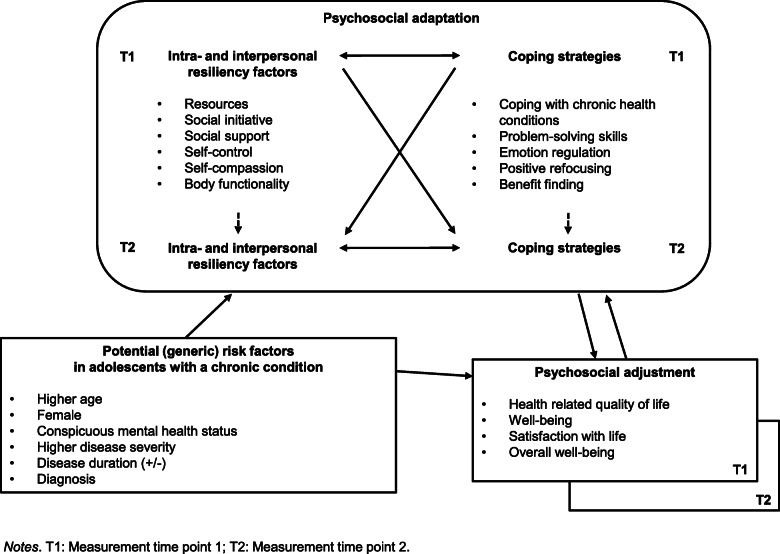
Conceptual model of the study

The primary aim of the project is to get a deeper understanding of the dynamic process of psychosocial adaptation, including intra- and interpersonal resiliency and coping strategies, in the face of CCs in a group of adolescents (12–21 years) with type 1 diabetes, cystic fibrosis, and juvenile arthritis. Hereby, psychosocial adaptation refers to the process of adaptation, while psychosocial adjustment refers to the outcome of this adaptation process [13, 24]. The research questions (Q) and hypotheses are as follows:

- Q1. How is the causal relationship between a) resiliency and coping strategies; b) psychosocial adaptation and adjustment? It is hypothesized that resiliency and coping strategies show reciprocal influences, and both precede the psychosocial adjustment.
- Q2. Does psychosocial adaptation (e.g., resilience factors and coping strategies) play a significant role in psychosocial adjustment beyond the influence of general demographic and CC-related factors? It is hypothesized that psychosocial adaptation is more important in explaining adjustment than the above-mentioned general factors (e.g., age, CC severity, gender). It is further assumed that females, as well as those showing conspicuous mental health screening, exhibit lower scores in psychosocial adjustment and therefore represent a high-risk group.
- Q3. Depending on general, demographic, and CC-related factors, do adolescents differ in psychosocial adaptation and adjustment? It is hypothesized that adolescents with higher age, female gender, more severe mental health problems and higher CC severity exhibit lower scores in psychosocial adaptation and adjustment.
- Q4. Depending on general, demographic, and CC-related factors, do adolescents differ in their course of psychosocial adaptation and adjustment over the one-year follow-up period? It is hypothesized that adolescents with higher age, female gender, more severe mental health problems and higher CC severity show a less favorable course of adaptation over the 1-year follow-up period compared to those with younger age, male gender, less severe mental health problems and lower CC severity. Figure 1 displays the conceptual model of the study.

## Methods/design

### Study design

**Fig. 2:**
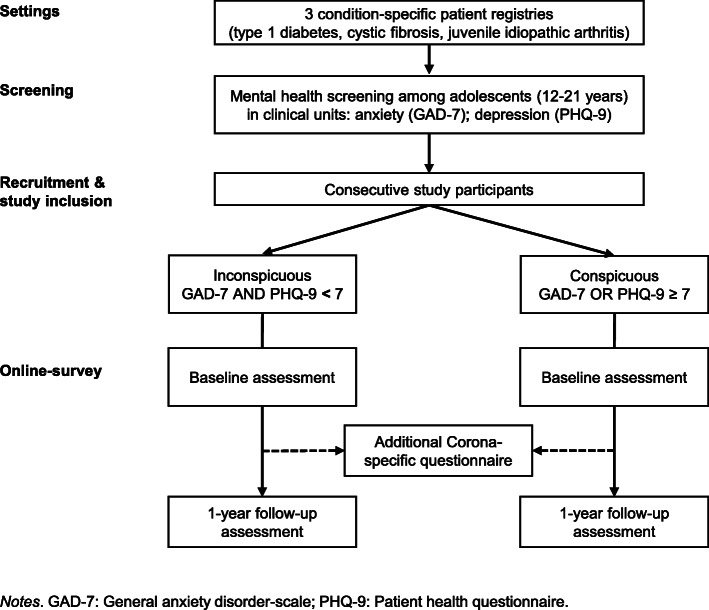
Study flow chart

This prospective observational study is part of a consortium (Chronic Conditions in Adolescents: Implementation and Evaluation of Patient-centered Collaborative Healthcare (COACH)) funded by the German Federal Ministry of Education and Research (no. 01GL1740C) that examines the mental health status of adolescents with a CC and examines different approaches to understand and overcome mental health issues [25, 26]. The study received approval by the Ethics Committee of the University of Potsdam (date 02/02/2018, no. 52/2017; date 08/31/2018, no. 37/2018; date 10/22/2018, reference no. 37/2018). Assessments take place starting with the mental health screening in clinical units, followed by two assessment time points over 1 year if adolescents agree to participate in the study: baseline (T1) and 12 months follow-up (T2). Based on the mental health screening for anxiety and depression, participants are assigned into the two parallel groups “inconspicuous” with scores below the cut-off (GAD-7 and PHQ-9 < 7) and “conspicuous” with scores above the cut-off (GAD-7 or PHQ-9 ≥ 7) [27–31]. During the recruitment we have been confronted with the Covid-19 pandemic and several regulations to contain the spread of the SARS-CoV-2 coronavirus. Therefore, we included an additional online survey to monitor the Covid-19 pandemic effects. The Covid-19-specific study received approval by the Ethics Committee of the University of Potsdam (date 04/29/2020, no. 27/2020; date 06/26/2020, reference no. 48/2020). The intermediate Covid-19-specific online survey was first implemented in April 2020 and sent out to all participants that had been included into the study up to this point. Since then, the questionnaire has been being sent to participants consecutively 1 week after completing the questionnaire at baseline (T1). The flow chart of the study design is shown in Fig. 2.

### Study participants

Adolescents between 12 and 21 years with type 1 diabetes, cystic fibrosis, or juvenile idiopathic arthritis are eligible for inclusion.

### Recruitment

Consecutive recruitment for this ongoing trial started in June 2019 in clinical centres. Adolescents with type 1 diabetes, cystic fibrosis, or juvenile idiopathic arthritis are recruited during their regular check-up visits in hospitals, clinics, medical practices, and medical centres across Germany. These clinical institutions all take part in one of the following German patient registries, the National Paediatric Rheumatologic Database (NPRD) [32], the National Diabetes Registry (DPV) [33], or the Cystic Fibrosis (CF) Registry [34]. Eligible patients (and their parents) are informed about the research project by the treating physician at the clinical centre and eligible patients are screened for anxiety and depression. All participants and parents of participants under legal age (< 18 years) provide written informed consent prior to inclusion into the study. Informed consent is sent to the University of Potsdam and all further recruitment activities are conducted by researchers there. In the next step, participants are invited to take part in the prospective online survey, which means that they are contacted by phone and receive an invitation email with the link to the survey. If they do not complete the questionnaire, they are contacted by email and phone at set time intervals and reminded to participate. Furthermore, they receive monthly SMS greetings to increase study adherence. To monitor the Covid-19 pandemic effects, participants are invited to take part in an intermediate online survey. Survey data will be connected with the objective medical background data provided by the patient registries: diagnosis, CC severity, CC duration and CC-specific data, e.g., hemoglobin A1c (HbA1c), forced expiratory volume in 1 s (FEV1), and clinical juvenile arthritis disease activity score (cJADAS). Principles of good research practice, data, and participants’ privacy protection are strictly adhered to. For each successfully recruited participant, the respective clinical unit receives a financial compensation of 75€ for its recruitment effort. Participants receive gift coupons as compensation for their time at T1 (20€) and T2 (30€). The online survey started in August 2019 and has been recruiting participants consecutively since. See Table 1 for preliminary descriptive data on the sample.

**Table 1:** Demographics of the preliminary sample

Variables	Total (***n*** = 426)	Inconspicuous (***n*** = 297)	Conspicuous (***n*** = 129)
Gender			
Male	185	141	44
Female	240	155	85
Non-binary	1	1	0
Age			
*M* (*SD*)	15.41 (2.05)	15.36 (2.03)	15.53 (2.11)
Range	12–21	12–21	12–21
Subjective socioeconomic status^a^			
*M* (*SD*)	6.73 (1.38)	6.92 (1.39)	6.30 (1.27)
Scale range^b^	Middle range	Middle range	Middle range
Chronic condition			
Type 1 diabetes	316	214	102
Cystic fibrosis	28	21	7
Juvenile idiopathic arthritis	82	62	20

*Note. M* Mean; *n* Number of participants; *SD* Standard deviation; Last day of data inclusion: 01/06/2021

^a^ Missings: *n* = 16

^b^ Scale ranges from 1 to 10

### Study measures

The comprehensive assessment in the observational study includes various psychological measures which are widely used in research. Unless otherwise stated, the name of each scale represents the underlying construct measured, with higher scores indicating more severe manifestations of the respective construct. Table 2 gives an overview over the study measures. References for study measures that have previously been published elsewhere are provided in the text (see Additional File 1 for self-constructed study measures).

**Table 2:** Study measures

Study Measures	T1	T2
**Psychosocial adjustment**		
DISABKIDS Chronic Generic Module (DCGM-37)	x	x
WHO-5 Well-Being Index (WHO-5)	x	x
Satisfaction with Life Scale (SWLS)	x	x
Self-Assessment Manikin (SAM)	x	x
**Psychosocial adaptation – Resources**		
Questionnaire of Resources in Childhood and Youth (FRKJ 8–16)	x	x
Questionnaire of Social Competence in Children and Adolescents, subscale social initiative (FSK-KJ)	x	x
Berlin Social Support Scales (BSSS), subscale seeking social support	x	x
Self-Control Scale (SCS-K-D)	x	x
Self-Compassion Scale (SCS-D short version)	x	x
Functionality Appreciation Scale (FAS)	x	x
**Psychosocial adaptation – Coping strategies**		
Coping with Chronic Health Conditions Questionnaire (CODI)	x	x
Questionnaire of Stress and Stress Management in Childhood and Adolescence (SSKJ 3–8), subscale problem-oriented coping	x	x
Emotion Regulation Questionnaire (ERQ)	x	x
Cognitive Emotion Regulation Questionnaire (CERQ), subscale positive refocusing	x	x
Benefit Finding Scale for Children (BFSC)	x	x
**Generic risk factors – Psychopathology**		
Patient Health Questionnaire (PHQ-9)	x^a^	x
General Anxiety Disorder-Scale (GAD-7)	x^a^	x
**Generic risk factors – Developmental tasks and stress**		
Questionnaire on Developmental Tasks in Adolescence		x
Questionnaire on critical life events		x
Perceived Stress Scale (PSS-4)		x
Questionnaire on overall impact of the Covid-19 pandemic		x
**Generic risk factors – Sociodemographic and CC-specific information**	x	x^b^

*Note.*^a^Assessed in clinical centres; ^b^Except age at diagnosis

### Assessment of adjustment: HRQoL and well-being

Multiple indicators for the assessment of HRQoL and well-being are used.

#### HRQoL

HRQoL is one of the major outcomes in medical research and is measured by the German version of the DISABKIDS Chronic Generic Module (DCGM-37) [35]. The 37 items are allocated to three domains with two facets each: psychological (independence, emotions), social (social exclusion, social inclusion), and physical (physical limitations, treatment/medication). The items are measured on a 5-point Likert scale ranging from 1 “never” to 5 “always”. A sample item is: “Do you have fun in your life?”. Internal consistency of the subscales ranges from Cronbach’s α = .70 to Cronbach’s α = .87. Internal consistency for the total score is Cronbach’s α = .93 [35].

#### Well-being

The German version of the 5-item WHO-5 Well-Being Index (WHO-5) assesses overall well-being [36]. Children and adolescents are asked to report the presence of positive feelings in the last 2 weeks on a 6-point scale ranging from 1 “at no time” to 6 “all the time” (e.g., “I have felt calm and relaxed.”). The WHO-5 can be validly administered to children and adolescents in pediatric care [37].

#### Satisfaction with life

The German version of the 5-item Satisfaction with Life Scale (SWLS) [38] is used to measure satisfaction with life (e.g., “I am satisfied with my life.”) whereby the first two items are adapted in their wording to the age group. The items are measured on a scale ranging from 1 “low satisfaction” to 7 “high satisfaction”. Internal consistency for the total score is Cronbach’s α = .92 [39].

#### Overall well-being

The Self-Assessment Manikin (SAM) is a picture-based assessment of affective reactions towards different stimuli [40, 41]. The current study focuses on overall well-being. Participants are asked “How do you feel in general?” and have to choose one of the five manikins ranging from looking very unhappy to smiling brightly.

### Assessment of psychosocial adaptation: resources and coping strategies

Multiple indicators for the assessment of psychosocial adaptation are used.

#### Resources in childhood and youth

Seven of the ten subscales of the Questionnaire of Resources in Childhood and Youth (FRKJ 8–16) are used to assess personal (empathy and perspective-taking, self-efficacy, self-esteem, optimism, and sense of coherence) and social resources (parental and social support, peer-group integration) in children and adolescents [42]. Each subscale contains six items. Participants’ responses are measured on a scale ranging from 1 “never true” to 4 “always true”. A sample item is: “When I set my mind to something, I get it done.”. The reliability of the subscales ranges from Cronbach’s α = .69 to Cronbach’s α = .89 [42].

#### Social initiative

The Questionnaire of Social Competence in Children and Adolescents (FSK-KJ) is a multidimensional questionnaire to assess social competencies in children and adolescents [43]. The current study uses the subscale “social initiative” (Cronbach’s α = .83) [43]. The subscale contains eight items with a response format ranging from 1 “not at all applicable” to 5 “absolutely applicable”. A sample item is: “I make friends easily.”.

#### Seeking social support

The Berlin Social Support Scales (BSSS) were developed in the context of coping with illness and measure social support on multiple dimensions [44]. The 5-item subscale “seeking social support” is used whereby participants rate how much the presented statements regarding support seeking apply to them on a scale ranging from 1 “strongly disagree” to 4 “strongly agree” (e.g., “When I have concerns, I seek out a conversation.”). In addition, the source of support is assessed by presenting a list of possible sources of support (e.g., parents, relatives, friends, teachers, etc.). Internal consistency of the total score is Cronbach’s α = .81 [44].

#### Self-control

The German version of the 13-item Self-Control Scale (SCS-K-D, short version) is used to assess dispositional self-regulatory behaviors (e.g., “I am good at resisting temptation.”) [45]. Items are scored on a 5-point Likert scale, ranging from 1 “not at all like me” to 5 “very much like me”. Internal consistency for the total score is Cronbach’s α = .79 [45].

#### Self-compassion

The German version of the Self-Compassion Scale (SCS-D, short version) encompasses 12 items rated on a 5-point scale, ranging from 1 “almost never” to 5 “almost always” [46] and comprises six subscales: self-kindness, self-judgment, common humanity, isolation, mindfulness, over-identification. A sample item is: “I try to be understanding and patient towards those aspects of my personality I don’t like.”. Internal consistency for the subscales ranges from Cronbach’s α = .54 to Cronbach’s α = .75 (total score: Cronbach’s α = .86) [47].

#### Body functionality

The German version of the 7-item Functionality Appreciation Scale (FAS) is used to assess body functionality [48]. Participants indicate their appreciation of body functionality on a 5-point Likert scale ranging from 1 “strongly disagree” to 5 “strongly agree” (e.g., “I appreciate my body for what it is capable of doing.”). Internal consistency for the FAS score is Cronbach’s α = .86 [48].

#### Coping with chronic health conditions

The German version of the 29-item Coping with Chronic Health Conditions Questionnaire (CODI) is used to assess coping strategies of children and adolescents with CCs [49]. The CODI comprises six domains: acceptance, avoidance, cognitive-palliative, distance, emotional reaction, and wishful thinking. Participants indicate their use of the coping strategies on a 5-point Likert scale ranging from 1 “never” to 5 “all the time” (e.g., “I try to ignore my illness.”). Depending on the domain, the internal consistency of the questionnaire ranges from Cronbach’s α = .69 to Cronbach’s α = .83 [49].

#### Problem-solving skills

Problem-solving skills are measured by the 6-item subscale “problem-oriented coping” of the Questionnaire of Stress and Stress Management in Childhood and Adolescence (SSKJ 3–8) [50]. The wording of the instruction was adapted to the specific CC context. Items are scored on a 5-point Likert scale, ranging from 1 “never” to 5 “always”. A sample item is: “I then decide on a way to solve the problem”. The internal consistency for the subscale is Cronbach’s α = 89 [50].

#### Emotion regulation

The German version of the 10-item Emotion Regulation Questionnaire (ERQ) is used to assess the habitual use of two emotion regulation strategies: cognitive reappraisal (6 items) and expressive suppression (4 items) [51]. Items are scored on a 7-point Likert scale, ranging from 1 “strongly disagree” to 7 “strongly agree”. A sample item is: “I keep my emotions to myself”. The subscales of the ERQ show an internal consistency of Cronbach’s α = .76 for the subscale “cognitive reappraisal” and Cronbach’s α = .74 for the subscale “expressive suppression” [51].

#### Positive refocusing

The Cognitive Emotion Regulation Questionnaire (CERQ) consists of nine subscales, each referring to what someone thinks after the experience of threatening or stressful events [52]. This study uses the German version of the 3-item subscale “positive refocusing “(e.g., “I think of pleasant things that have nothing to do with it.”). Participants indicate on a 5-point Likert scale ranging from 1 “(almost) never” to 5 “(almost) always” how much they rely on positive refocusing. The internal consistency of the subscale is Cronbach’s α = .85 [53].

#### Benefit finding

A German version of the 10-item Benefit Finding Scale for Children (BFSC) is used to assess benefit finding [54]. Participants indicate the extent to which their illness has helped them grow personally (e.g., “Has helped me to become a stronger person.”). Their responses are measured on a 5-point scale ranging from 1″ not at all true for me” to 5 “very true for me”. The BFSC has an internal consistency of Cronbach’s α = .83 [54].

### Assessment of psychopathology

Additional instruments were used at T2, including the PHQ-9 and the GAD-7 from the mental health screening in the clinical centres to assess development of mental health of the participants.

#### Depression

The German version of the 9-item Patient Health Questionnaire (PHQ-9) is administered as a screening inventory to detect depressive symptoms within the past 2 weeks (e.g., “Little interest or pleasure in doing things.”) [55]. We omitted one item assessing suicidal ideation. Responses are measured on a 4-point Likert scale ranging from 0 “not at all” to 3 “nearly every day”. The computerized version of the PHQ-9 has an internal consistency of Cronbach’s α = .88 [56].

#### Anxiety

The German version of the General Anxiety Disorder-Scale (GAD-7) is administered to detect symptoms of anxiety within the past 2 weeks (e.g., “Feeling nervous, anxious or on edge.”) [57, 58]. The GAD-7 consists of seven items with a 4-point Likert scale ranging from 0 “not at all” to 3 “nearly every day”. The GAD-7 shows an internal consistency of Cronbach’s α = .79 to Cronbach’s α = .91 [59] and successful usage in adolescents is reported [60, 61].

### Assessment of developmental tasks and stress

Multiple indicators for the assessment of developmental tasks and stress are used.

#### Developmental tasks

A modified version of the Questionnaire on Developmental Tasks in Adolescence is administered to assess normative developmental tasks in youth [62]. For the current study, the questionnaire was shortened to eleven items and the wording was adapted to the targeted age group. Participants indicate their subjective importance of the presented developmental tasks on a 6-point Likert scale ranging from 1 “not (yet) important” to 6 “no longer important” (e.g., become more independent from parents, accepting changes in the body and one’s appearance). Participants also mark on a line where they are in the process of completing each developmental task.

#### Critical life events

A self-constructed 10-item questionnaire assesses critical life events that occurred in participants’ life during the past 12 months (e.g., “Did you move out of your parents’ house?”). If participants select one or more of the events, they are asked to indicate how each of them has impacted their lives on a slider ranging from 0 “not at all” to 100 “completely”. Additionally, an open format question asks for further potential life events not covered by the items presented.

#### Perceived stress

The German version of the 4-item Perceived Stress Scale (PSS-4) is used to assess the frequency of perceived stress in the past 12 months (e.g., “In the last twelve months, how often have you felt that you were unable to control the important things in your life?”) [63]. The items are measured on a 5-point Likert scale ranging from 1 “never” to 5 “very often”. The internal consistency is Cronbach’s α = .72 [63].

#### Overall impact of the Covid-19 pandemic

A self-constructed 4-item Covid-19-specific scale is administered to assess the impact of the Covid-19 pandemic on different life domains: family life, education, interaction with friends and leisure activities. Participants use a slider to indicate how much each of these life domains has changed as a result of the Covid-19 pandemic on a scale ranging from 0 “not at all” to 100 “completely”.

### Sociodemographic and CC-specific information

Sociodemographic information collected in the study comprise gender, age, body size and weight, living situation, and education as self-assessments [64]. Subjective socioeconomic status is assessed by a slightly modified version of the MacArthur Scale [65]. CC-specific variables encompass the CC itself, age at diagnosis, further physical and mental disorders, psychological treatment, subjective severity of the CC, subjective health status using three items that were developed based on widely used measures [66, 67], as well as satisfaction with treatment staff and identification with the group of people who have the same CC [68].

### Covid-19 monitoring

In reaction to the Covid-19 pandemic, the additional online survey contains questions regarding psychosocial adjustment and adaptation as well as risk perception, emotions, psychological distress and the impact of the Covid-19 pandemic. Established instruments, as well as self-constructed Covid-19-specific instruments, are used for this purpose (see Table 3 for an overview and Additional file 2 for detailed information on the Covid-19 specific measures).

**Table 3:** Study measures for intermediate Covid-19 study

Study Measures	Covid-19 study
**Psychosocial adjustment**	
WHO-5 Well-Being Index (WHO-5)	x^a^
Satisfaction with Life Scale (SWLS)	x^a^
**Psychosocial adaptation – Resources**	
Questionnaire of Resources in Childhood and Youth (FRKJ 8–16), subscale peer-group integration	x
Berlin Social Support Scales (BSSS), subscale seeking social support	x^b^
**Psychosocial adaptation – Coping strategies**	
Benefit Finding Scale for Children (BFSC)	x^b^
**Covid-19-specific resources**	
Self-efficacy beliefs	x
Outcome expectations	x
Resilience	x
Contact to peers	x
Social norms	x
**Covid-19-specific coping strategies**	
Coping across Situations Questionnaire (CASQ)	x^b^
Preparedness	x
**Risk perception**	x
**Emotions and psychological distress during the Covid-19 pandemic**	
Positive and Negative Affect Schedule (PANAS)	x
Perceived Stress Scale (PSS-4)	x
Emotions	x
**General questions regarding the Covid-19 pandemic**	x
**Overall impact of the Covid-19 pandemic**	x^c^

*Note.*^a^As used in T1 and T2. ^b^Adapted to Covid-19 pandemic situation. ^c^Includes additional items

### Sample size

The sample size for the prospective analysis is determined by the main and most complex analysis applying latent structural equation modeling (SEM). Up to now, there is no unambiguous rule on required sample sizes in SEM, as it is dependent on several aspects (e.g., model complexity, data distribution, amount of missing data, effect size, and number of parameters that have to be computed). As a rule of thumb, the sample size should not be lower than 200 although computations should be possible with sample sizes greater than 100 [69]. As a multi-group latent SEM (grouping factor: mental health status inconspicuous vs. conspicuous) should be applied to the data, a total sample size of 400 should be sufficient. Both the inconspicuous and the conspicuous group should consist of a minimum of 100 participants. Therefore, at least 200 participants in each group should be achieved. However, regarding the prevalence of depression and anxiety in youth with CCs, while considering possible barriers to participate with symptoms of depression and/or anxiety, we expect a higher amount of inconspicuous participants. To reduce bias in estimates obtained, which may occur due to non-random differences in covariates between groups, we will consider appropriate matching procedures. Therefore, we increased the targeted sample size for the inconspicuous group to 250 participants during the recruitment process.

### Statistical analyses

Prior to analyses, we will exclude participants based on the following criteria to decrease noise in online survey data: relative speed indicator higher than 2, which means that the respondent has completed a page twice as fast as a typical respondent [70], and a completion time < 5 min, which means an unrealistic absolute completion time. To deal with missing data and drop-out, multiple imputations via fully conditional specification implemented by the MICE algorithm will be used [71].

We will apply cross-lagged panel models to estimate the reciprocal relationships between a) resiliency and coping strategies and b) psychosocial adaptation and adjustment. Dependent measures will be checked for normality and then centered based on their grand mean. As a first step, we will conduct longitudinal confirmatory factor analyses (CFA) for each cohort (“inconspicuous”; “conspicuous”), using the covariance matrix. In a second step, we will specify longitudinal cross-lagged panel models with a) resiliency and coping strategies and b) psychosocial adaptation and adjustment as latent factors for each group. In a third step, we will conduct a multigroup analysis, which combines the models for both cohorts. In a fourth step, we will implement the covariates (e.g., age, gender) into the final models. In a final step, we will split latent predictors into their respective indicators examining the relations between a) resiliency and coping strategies and b) psychosocial adaptation and adjustment at the level of components.

Regression analyses will be applied to analyze the incremental/additional contribution of adaptation in psychosocial adjustment beyond the influence of general demographic and CC-related factors. HRQoL (total score and subscales’ scores) will serve as dependent variable(s), the sociodemographic, CC-specific risk factors and the psychosocial adaptation will form the independent variables. The analysis on differences in a) resiliency and coping strategies and b) the psychosocial adaptation and adjustment depending on demographic and CC-related factors will be addressed by three multifactorial ANOVAs. To examine differences in the course of psychosocial adaptation and psychosocial adjustment, multifactorial repeated measures ANOVAs will be applied. Before these analyses (Hypotheses 2–4), data will be checked on homoscedasticity, normal distribution of errors and multicollinearity. We will control for overall Covid-19 related distress for all analyses, if there is a significant association with psychosocial adjustment at T1 and/or T2. The analyses will be conducted using R [71–74].

## Discussion

CCs are quite common among adolescents and can hinder their age-appropriate psychosocial development. So far, research has mainly focused on various risk factors for such an unfavorable development. The present study aims to undertake a paradigm shift in the research on CCs by explicitly focusing on the intrapersonal resources of those affected and a broad range of psychosocial adjustments. The present study aims to examine the reciprocal associations between resiliency and coping strategies (psychosocial adaptation) on the one hand and psychosocial adjustment on the other hand across a one-year period in a sample of adolescents facing CCs. The findings will expand our current knowledge addressing the following aspects: (1) This study focuses on an assessment of resiliency factors and coping strategies in explaining psychosocial adjustment. Up to now, protective factors and their incremental contribution have been rarely addressed but provide unique starting points for preventional and interventional approaches; (2) A major strength is the inclusion and longer-term follow-up of youths in a sensitive developmental stage for the emergence of mental health problems, non-adherence and health-related risk behaviors [22]. Adolescence is considered to be the “cradle of health-related behaviors” and therefore provides great opportunities for maintaining and improving current and future health [22]; (3) The simultaneous consideration of specific CCs differing in various CC-related aspects (e.g., the role of pain; medical treatment or life-expectancy) follows a non-categorical approach [75]. By including CCs with diverse characteristics, one can identify the unique vs. generic characteristics in the process of psychosocial adaptation. Especially the identification of generic modifiable aspects will support the theory-driven development of non-categorical interventions, which is highly important for those adolescents with a rare condition [7]. (4) The broad recruitment via the German patient registries enables a representative sample. (5) We will include both, those adolescents facing problems in coping with their CC and those who are doing well. By ensuring that both subsamples are nearly comparable with respect to the sample size, we will be able to compare their respective resource profiles. In addition, the sample sizes will be large enough for applying multi-group comparisons over time. (6) Using a comprehensive set of instruments, we can assess various aspects of psychosocial adaptation in the face of CCs (e.g., social support, self-compassion, and problem-solving skills). (7) The study takes an interdisciplinary approach: Medical data are linked to psychological parameters, thus allowing deeper insights into potential resilience effects on medical health parameters among others.

Possible obstacles and limitations are as follows: (1) A prospective study design bears the risk of a higher drop-out, especially of those who show higher levels of emotional distress [76]. To minimize the risk of a selective drop-out, participants receive a monetary incentive, which will likely increase study adherence. In addition, we have established regular contact via short text messages over the course of the observation period to reinforce committment to the study [77]. (2) The Covid-19 pandemic and particularly the first lockdown had an influence on our recruiting success because medical appointments were postponed or patients did not show up, some medical facilities had to take on other medical care tasks, etc. Furthermore, the Covid-19 pandemic posed an additional coping challenge for youths and will most likely have an impact on their psychosocial adaptation, as the pandemic elicits life events with uncertainty, ambiguity, and loss of control, each of which is known to trigger psychosocial distress, including anxiety, depression, and anger [78]. Especially individuals with CCs might experience more emotional distress than their “healthy” peers, as they are at high risk for severe Covid-19 disease progress [79]. It can be assumed that intra- and interpersonal resiliency factors also have a major influence on the adaptation when dealing with this situation. Therefore, we decided to monitor possible Covid-19 pandemic effects by implementing an additional Covid-19 assessment. This will allow us to observe coping with a stressful life event and to examine its unique influence.

In conclusion, our study will contribute to a deeper understanding of the dynamic process of psychosocial adaptation when facing a CC. Based on the empirical data of this longitudinal observational study, tailored resource-based interventions supporting a positive adaptation in youths with a CC can be developed. Such intervention approaches are not only suitable for helping those adolescents who already face adjustment problems. They can also be applied to prevent them from developing in the first place.

## Supplementary Information

**Additional file 1:.** Self-constructed study measures.

**Additional file 2:.** Measures for assessment of Covid-19-related factors.

## Data Availability

Fully anonymized data will be available from the corresponding author on reasonable request and with the permission of the collaboration partners.

## Abbreviations

BFSC: Benefit finding scale for children
BSSS: Berlin social support scales
CC: Chronic condition
CERQ: Cognitive emotion regulation questionnaire
CF: Cystic fibrosis
CFA: Confirmatory factor analyses
cJADAS: Clinical juvenile arthritis disease activity score
COACH: Chronic conditions in adolescents: Implementation and evaluation of patient-centered collaborative healthcare
CODI: Coping with chronic health conditions
DCGM-37: DISABKIDS chronic generic module
ERQ: Emotion regulation questionnaire
DPV: National diabetes registry
FAS: Functionality appreciation scale
FEV1: Forced expiratory volume in 1 s
FRKJ 8–16: Questionnaire of resources in childhood and youth
FSK-KJ: Questionnaire of social competences in children and adolescents
GAD-7: General anxiety disorder-scale
HbA1c: Hemoglobin A1c
HRQoL: Health-related quality of life
NPRD: National paediatric rheumatologic database
PHQ-9: Patient health questionnaire
PSS-4: Perceived stress scale
Q: Question
SAM: Self-assessment manikin
SCS-D: Self-compassion scale
SCS-K-D: Self-control scale
SEM: Structural equation modeling
SSKJ 3–8: Questionnaire of stress and stress management in childhood and adolescence
SWLS: Satisfaction with life scale
WHO-5: WHO-5 well-being index
T1: Measurement time point 1
T2: Measurement time point 2
