# Supplementary material for: A prospective investigation of developmental trajectories of psychosocial adjustment in adolescents facing a chronic condition - study protocol of an observational, multi-center study
Source: BMC Pediatr. 2021 Sep 14;21:404. doi: 10.1186/s12887-021-02869-9 (PMC8438102; doi:10.1186/s12887-021-02869-9)
Supplement: Supplementary file 1 — Additional file 1:. Self-constructed study measures. [file 12887_2021_2869_MOESM1_ESM.docx]

**Additional file 1**. Self-constructed study measures

**Self-constructed study measures for main study**

***Critical life events***

A lot can happen in 12 months. We have compiled a series of possible life events. Check off what happened to you in the past year.

1. Have you experienced any change in your education, career or training (e.g., change of school, career or study subject, repetition of a class, expulsion from school, the start of training/studies, etc.)?
2. Have you moved out of your parents' house?
3. Did you fall in love and start a relationship, or did your relationship end?
4. Were there complications in connection with your chronic illness (e.g., unexpected hospitalization)?
5. Have you changed the doctor you used to see for your chronic condition?
6. Have you had a serious illness or accident?
7. Has a family member or close friend of yours had a serious illness or accident?
8. Has someone close to you died?
9. Did your parents separate?
10. Has there been another positive or negative life event? If yes, which one?
11. Was there another positive or negative life event? If yes, which one?

***Overall impact of the Covid-19 pandemic***

Please indicate how much the following areas of your life have changed because of the Covid-19 pandemic:

- Family life
- School/training/studies
- Interaction with friends
- Leisure activities

**Self-constructed study measures for intermediate Covid-19 study**

***Outcome expectations***

We would like to know your attitude towards the new rules of conduct in dealing with the SARS-CoV-2 coronavirus.

When I follow the rules for social distancing, ...

1. ... I can reduce my risk of infection.
2. ... I can spend less time with my friends. (reversed item)
3. ... my family disapproves. (reversed item)
4. ... my friends disapprove. (reversed item)
5. ... it is good for the health of others.
6. ... it means a loss of quality of life for me. (reversed item)
7. ... I am a good role model for my family.
8. ... I am a good role model for my friends.
9. ... I am less susceptible to illness.

***Contact to peers***

What about your friends?

1. How often do you meet with your friends in person right now?
2. How often did you meet your friends in person before the pandemic?
3. How often are you in touch with your friends by phone or digital media (e.g., Whatsapp, Facebook, Instagram, Skype, or similar)?
4. Before the pandemic, how often were you in contact with your friends by phone or via digital media (e.g., Whatsapp, Facebook, Instagram, Skype or similar)?

***Social norms***

What is it like with your family and friends?

1. In my **family**, it is considered important to follow the rules for regular hand washing.
2. In my **group of** **friends**, it is considered important to follow the rules for regular hand washing.
3. In my **family**, it is considered important to keep the rules about social distance.
4. In my **group of friends,** it is considered important to keep the rules of social distance.
5. In my **family**, the rules for regular hand washing are considered exaggerated. (reversed item)
6. Among my **friends**, the rules for regular hand washing are considered exaggerated. (reversed item)
7. In my **family**, the rules about social distance are considered exaggerated. (reversed item)
8. Among my **friends**, the rules about social distance are considered exaggerated. (reversed item)

***Preparedness***

Finally, we would like to know to what extent you feel prepared for the new rules of conduct in dealing with the new SARS-CoV-2 coronavirus.

1. Coping with my disease on a day-to-day basis helped me to deal with the current contact ban better than other adolescents.
2. My disease has taught me to cope with restrictions better than other adolescents.
3. Compared to other adolescents, I am better able to cope with the current rules of conduct.
4. It is easier for me to keep in touch with my friends in the current situation than usual.
5. Since everyone is currently communicating mainly via cell phone or digital media, I can participate in as many social activities as other young people.

***Perceived distress***

How much is the current Covid-19 pandemic stressing you out?

**Overall impact of the Covid-19 pandemic**

1. Please indicate how much the following areas of your life have changed as a result of the Covid-19 pandemic:

- Family life
- School/training/studies
- Interaction with friends
- Leisure activities
- Coping with your condition

1. How well informed do you feel about available measures?
2. How do you feel about the situation?
3. Can you briefly describe how the Covid-19 pandemic affects your personal daily life?
4. Can you briefly describe how the Covid-19 pandemic affects the way you deal with your illness?
5. You have been told by many sources (media, government, your parents, etc.) that you should limit social contact. What convinced you personally that it is important to limit social contact at this time?
