# Supplementary material for: A prospective investigation of developmental trajectories of psychosocial adjustment in adolescents facing a chronic condition - study protocol of an observational, multi-center study
Source: BMC Pediatr. 2021 Sep 14;21:404. doi: 10.1186/s12887-021-02869-9 (PMC8438102; doi:10.1186/s12887-021-02869-9)
Supplement: Supplementary file 2 — Additional file 2:. Measures for assessment of Covid-19-related factors. [file 12887_2021_2869_MOESM2_ESM.docx]

**Additional file 2**. Measures for assessment of Covid-19-related factors

Unless otherwise stated, the name of each scale represents the underlying construct measured, with higher scores indicating more severe manifestations of the respective construct. Some of the instruments are adapted in their wording to the adolescent sample. References for study measures that have previously been published elsewhere are provided in the text (see Additional File 1 for self-constructed study measures).

**Covid-19-specific resources**

***Self-efficacy beliefs***

Items from the short version of the German version of the 6-item Generalized Self-Efficacy scale (GSE) were adapted to assess Covid-19-specific self-efficacy beliefs in adolescents [1,2]. Participants indicate on a 7-point Likert scale ranging from 1 “not at all true” to 7 “exactly true” how well they are handling everyday problems during the current Covid-19 pandemic (e.g., “I easily find ways to stay in touch with my friends without meeting them.”).

***Outcome expectations***

A self-constructed 9-item questionnaire based on the HAPA-model assesses outcome expectations [3]. Participants indicate on a 7-point Likert scale ranging from 1 “not at all true” to 7 “exactly true” their attitude toward the new behavioural rules for dealing with the SARS-CoV-2 coronavirus (e g. “If I follow the rules on social distancing, I will be able to reduce my risk of infection.”).

***Resilience***

Four Items from the COSMO-project were adapted to assess Covid-19-specific resilience [4]. Participants indicate on a 7-point Likert scale ranging from 1 “not at all true” to 7 “exactly true” how resilient they are in the face of the Covid-19 pandemic (e.g., “During the Corona Pandemic, I am learning important and useful lessons for my life.”).

***Contact to peers***

A self-constructed 4-item questionnaire assesses the amount of social contact to peers before and during the pandemic (e.g., “How often are you meeting with your friends in person right now?”). Items are measured on a 5-point Likert scale ranging from 1 “very rarely” to 5 “very often”.

***Social norms***

A self-constructed 8-item questionnaire assesses social norms, specifically the extent to which friends and family are conforming to the Covid-19-related rules about social distance and washing hands regularly. Participants indicate on a 7-point Likert scale ranging from 1 “not at all true” to 4 “neutral” to 7 “exactly true” how well friends and family are complying with Covid-19-related social norms.

**Covid-19-specific coping strategies**

***Coping***

The German version of the 20-item Coping across Situations Questionnaire (CASQ) was adapted to assess Covid-19-specific coping [5]. The items are allocated to two domains (active coping and problem-avoiding behaviors), whereby item 6 was omitted for content reasons in this study. Participants indicate on a 5-point Likert scale ranging from 1 “never” to 5 “always” how they are dealing with day-to-day issues during the Covid-19 pandemic (e.g., “In the current situation, I'm bracing myself for the worst.”).

***Preparedness***

A self-constructed 5-item questionnaire assesses preparedness. Participants indicate on a 7-point Likert scale ranging from 1 “not at all true” to 7 “exactly true” the extent to which they feel prepared to handle the SARS-CoV-2 coronavirus compared with healthy adolescents (e.g., “Compared to other adolescents, I am able to cope better with the current rules of conduct.”).

**Risk perception**

Six slightly modified items from the COSMO-project assess subjective risk perception of adolescents, specifically their own risk, their own risk compared to other adolescents, their risk for severe infection with the SARS-CoV-2 coronavirus and their risk for severe infection with the SARS-CoV-2 coronavirus compared to other adolescents [4]. Items assessing their own risk are measured on a 7-point Likert scale ranging from 1 “very unlikely” to 7 “very likely”. Items assessing their own risk compared to other adolescents are measured on a 7-point Likert scale ranging from 1 “significantly lower” to 7 “significantly higher”. Items assessing their risk for a severe infection with SARS-CoV-2 coronavirus are measured on a 7-point Likert scale ranging from 1 “completely harmless” to 7 “very harmful”. Items assessing their risk for a severe infection with SARS-CoV-2 coronavirus compared to other adolescents are measured on a 7-point Likert scale ranging from 1 “significantly more harmless” to 7 “significantly more harmful”.

**Emotions and psychological distress during the Covid-19 pandemic**

***Positive/negative affect***

A slightly modified German version of the 20-item Positive and Negative Affect Schedule (PANAS) assesses positive and negative affect in children and adolescents (e.g., “feeling active” or “feeling fearful”) [6]. The items are measured on a 5-point Likert scale ranging from 1 “not at all” to 5 “extremely”. The internal consistency of the scale is α ≥ .84 [6].

***Perceived distress***

A self-constructed item assesses perceived distress. Participants indicate, using a slider ranging from 0 “not at all” to 100 “completely”, how much the current Covid-19-pandemic is stressing them out.

***Emotions***

Five items from the COSMO-project assess the emotional reaction to the SARS-CoV-2 coronavirus [4]. The items are measured on a 7-point semantic differential scale ranging from 1 “negative emotion” to 7 “positive emotion” (e.g., “scary” to “not scary”).

**General questions regarding the Covid-19** **pandemic**

Three slightly modified items from the COVID-19 Snapshot Monitoring-project (COSMO-project) assess whether participants themselves or people around them were infected with the SARS-CoV-2 coronavirus and whether participants were in a health department-imposed quarantine [4]. Furthermore, a self-constructed item assesses whether upcoming or already scheduled medical appointments for the treatment of the given chronic disease had been cancelled.

**Overall impact of the Covid-19 pandemic**

A self-constructed 4-item Covid-19-specific scale is administered to assess the impact of the Covid-19 pandemic on different life domains: family life, education, interaction with friends, leisure activities, and coping with the chronic condition. The participants use a slider to indicate how much the areas of their life in question have changed as a result of the Covid-19 pandemic on a scale ranging from 0 “not at all” to 100 “completely”. Additionally, participants indicate how well informed they feel about available measures and how they are doing in the current situation using a slider on a scale ranging from 0 “very poorly” to 100 “very well”. Furthermore, three self-constructed open-ended items assess how the Covid-19 pandemic affects daily life and coping with the condition and examine what convinces participants to limit their social contact (e.g., “Can you briefly describe how the Corona pandemic affects your personal day-to-day life?”).

**Abbreviations**

CASQ: Coping across situations questionnaire; COSMO-project: COVID-19 Snapshot Monitoring-project; GSE: Generalized self-efficacy; PANAS: Positive and negative affect schedule.

**References**

1. Romppel R, Herrmann-Lingen C, Wachter R, Edelmann F, Düngen HD, Pieske B, Grande G. A short form development of the General Self-Efficacy Scale (GSE-6): De-velopment, psychometric properties and validity in an intercultural non-clinical sample and a sample of patients at risk for heart failure. GMS Psycho-Social-Medicine 2013;10:1-7.

2. Schwarzer R, Jerusalem M. Generalized Self-Efficacy scale. In: Weinman J, Wright S, Johnston M. Measures in health psychology: A user’s portfolio. Causal and control be-liefs. Windsor: NFER-NELSON; 1995. pp. 35-37.

3. Renner B, Schwarzer R. Risiko und Gesundheitsverhalten. Dokumentation der Mess-instrumente des Forschungsprojekts „Risk Appraisal Consequences in Korea“ (RACK) 2003. Berlin: FU Berlin; 2005.

4. Betsch C, Korn L, Felgendreff L, Eitze S, Schmid P, Sprengholz P, Wieler L, Schmich P, Stollorz V, Ramharter M, Bosnjak M, Omer SB, Thaiss H, De Bock F, Von Rüden U, Lieb K, Thrull J, Lämmlin G. German COVID-19 snapshot monitoring (COSMO) - Welle 4. PsychArchives. 2020;1-74; https://doi.org/10.23668/PSYCHARCHIVES.2862.

5. Seiffge-Krenke I, Shulman S. Coping style in adolescence: A cross-cultural study. J Cross Cult Psychol. 1990;21(3):351-377.

6. Breyer B, Bluemke M. Deutsche Version der Positive and Negative Affect Schedule PANAS (GESIS Panel). Zusammenstellung sozialwissenschaftlicher Items und Skalen (ZIS). 2016; https://doi.org/10.6102/zis242.
